# Efficacy and safety of electro-acupuncture treatment in improving the consciousness of patients with traumatic brain injury: study protocol for a randomized controlled trial

**DOI:** 10.1186/s13063-018-2687-3

**Published:** 2018-05-29

**Authors:** Jie Liu, Xinsheng Xue, Ying Wu, Chaohua Yang, Ning Li, Huiping Li

**Affiliations:** 1 Department of Traditional Chinese Medicine and Western Medicine, West-China Hospital of Sichuan University, Guoxuexiang 37, Chengdu, 610041 Sichuan China; 2 Department of Critical Care Medicine, West-China Hospital of Sichuan University, Guoxuexiang 37, Chengdu, 610041 Sichuan China; 3 Department of Neurology, West-China Hospital of Sichuan University, Guoxuexiang 37, Chengdu, 610041 Sichuan China; 4 Department of Neurosurgery, West-China Hospital of Sichuan University, Guoxuexiang 37, Chengdu, 610041 Sichuan China

**Keywords:** Electro-acupuncture, Traumatic brain injury, Consciousness, Mismatch, Negativity

## Abstract

**Background:**

Traumatic brain injury (TBI) has become a leading cause of death among young people worldwide. Survivors may live with a long-term TBI-related disability or even develop a disorder of consciousness resulting in poor life quality and shortened life expectancy. Thus far, very few approaches have been found to be effective in the consciousness recovery of these patients. Acupuncture has long been used in the treatment of neurological disorders in China. However, its efficacy and safety in consciousness recovery remain to be proved.

**Methods:**

Here, we present a study design and protocol of a randomized, blinded, controlled study to evaluate the efficacy and safety of electro-acupuncture in the consciousness recovery of patients with TBI. A total of 150 patients with initial Glasgow coma scale score of less than 8 points will be recruited in the trial and randomized into acupuncture or control groups. Patients in the control group will receive routine pharmacological treatment alone while patients in the acupuncture group will receive electro-acupuncture treatment for 10 days in addition to routine treatment. The efficacy will be assessed with the changes in Glasgow coma scale score and mismatch negativity of event-related brain potentials before and after treatment. Moreover, Glasgow outcome scale and Barthel index of activities of daily living will be compared between the two groups at 3 months after treatment. The secondary outcome measures are the length of stay in ICU and hospital, expenses in ICU and hospital, as well as the incidence of coma-related complications. The safety of electro-acupuncture will be assessed by monitoring the incidence of adverse events and changes in vital signs during the study.

**Discussion:**

Results from this trial will significantly add to the current body of evidence on the role of electro-acupuncture in the consciousness recovery of patients with severe TBI. In addition, a more convenient and consistent electro-acupuncture method can be set up for clinical practice. If found to be effective and safe, electro-acupuncture will be a valuable complementary option for comatose patients with TBI.

**Trial registration:**

Chinese Clinical Trial Registry: ChiCTR-INR-17011674. Registered on 16 June 2016.

**Electronic supplementary material:**

The online version of this article (10.1186/s13063-018-2687-3) contains supplementary material, which is available to authorized users.

## Background

Traumatic brain injury (TBI) is a major cause of mortality and morbidity in young people [1]. Despite the continuous advances in neurosurgery, numerous survivors suffer from TBI-related disabilities. It is estimated that 5.2 million and 7.3 million people in the USA and in the European Union, respectively, have disabilities due to TBI [2, 3]. Given the rapid development of urban construction and transportation, the incidence of TBI in China has increased dramatically in recent years, with the current mortality rate within the range of 2.7% to 21.8% [4]. Due to the limited healthcare and rehabilitation sources, TBI survivors depend mainly on their families for their treatment, especially those who fail to recover consciousness. Therefore, the TBI-related disability of survivors has become a critical public health issue in China.

Neurocognitive deficits are common in patients with severe TBI. Patients who fail to fully recover consciousness may enter a disorder of consciousness (DOC) such as coma, a vegetative state, or a minimally conscious state [5]. With the development of diagnostic tools such as functional magnetic resonance and electroencephalography, clinicians are able to detect DOC in patients with severe TBI and commence early intervention [6, 7]. Current therapeutic strategies for DOC include behavioral, pharmacological, and neurostimulatory approaches, among which the latter two have been intensively studied. For example, amantadine and other dopaminergic agents have shown beneficial effects in promoting recovery after TBI, but warrant further systemic investigation [8–10]. As a potential restorative treatment for DOC, neurostimulation has attracted increasing interest. A study of thalamic deep brain stimulation demonstrated a remarkable behavioral recovery in patients with TBI [11]. Non-invasive forms of neurostimulation, such as transcranial magnetic stimulation and transcranial direct current stimulation, have shown some short-term effects on behavioral improvement, but need further study [12–14].

Acupuncture therapy is an important component of traditional Chinese medicine and has long been used in China to promote the neurological functions in patients with stroke and hemiplegia [15]. Although numerous studies have been conducted to evaluate the effects of acupuncture, results from these randomized controlled trials are controversial due to problems concerning the study design, including small sample size, high drop-out rate, lack of proper control, and randomization [16].

Herein, we present a study design and protocol of a randomized, blinded, controlled study to evaluate the efficacy and safety of electro-acupuncture in improving the consciousness level in patients with TBI. This study aims to provide high-quality evidence for the application of acupuncture in the management of comatose patients as well as to establish a standard acupuncture procedure for clinical practice.

## Methods

### Design and setting

This is a single-center, randomized controlled trial. This study has been approved by the Ethical and Biomedical Research Committee of West-China Hospital, Sichuan University. It is registered on http://www.chictr.org.cn with registration number ChiCTR-INR-17011674.

The study will be conducted in the West-China Hospital of Sichuan University. The legal representative of each patient will be asked to sign an informed consent form. To avoid selective bias, all enrolled patients will be divided into two equal groups to receive either acupuncture plus routine treatment (acupuncture group) or routine treatment alone (control group) using the random scheme generated from SPSS software. The serial number will be sealed in a non-transparent envelope by the principal investigator and given to the researcher. Acupuncture will be performed by doctors from the Department of Traditional Chinese and Western Medicine. The efficacy and safety will be evaluated by doctors from the Department of Critical Care Medicine, Department of Neurosurgery and Department of Neurology, who will be blind to the allocation of the patients.

### Participants

A total of 150 patients with TBI being treated at West-China Hospital of Sichuan University will be enrolled in this study after the legal representative signs the informed consent. The inclusion, exclusion, and withdrawal criteria are described in Table 1.

**Table 1:** Inclusion and exclusion criteria for electro-acupuncture in severe traumatic brain injury trial

Inclusion	Patients with severe traumatic brain injury admitted to ICU: • Initial GCS score less than 8 points on admission • Estimated ICU stay longer than 2 weeks • Normal consciousness level before brain injury, no primary disorders in cognitive or motor function • Aged from 18 to 75 years • Agree to participate in the study and sign the informed consent
Exclusion	Patients are excluded from the study if any of the following criteria apply: • Vital signs are not stable on admission, die within 24 h • Women with pregnancy • Legal representative or immediate family has no strong treatment intent, can withdraw from treatment within 1 week • Patients with multiple trauma associated with limb fracture or skin defect
Withdrawal	Patients will be withdrawn from the study if any of the following criteria applies: • Life-threatening complications • Secondary hydrocephalus • Uncontrolled intracranial infection • Re-operate due to varieties of causes • Massive cerebral infarction • Unable to follow-up

### Procedure

**Fig. 1:**
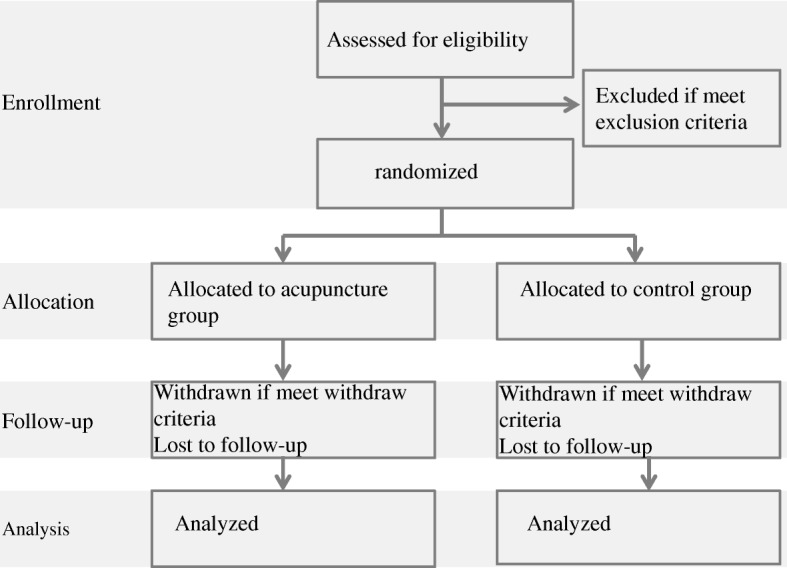
Flow chart of the study procedure

Figure 1 shows the study procedure flowchart. Potential participants from the Department of Critical Care Medicine, Shangjin clinics, West-China Hospital of Sichuan University, will be enrolled according to the inclusion criteria and randomized into the acupuncture or control groups. In addition to routine treatment, patients in the acupuncture group will receive a 10-day electro-acupuncture treatment. Acupuncture will start 3 days after trauma if the patient’s situation is stable. Patients in the control group will receive routine treatment alone, involving the prescription of coma arousal and neuroprotective agents by the neurosurgeons. If the patient needs vasoconstrictor or hypothermia therapy due to an unstable intracranial situation, electro-acupuncture will be started 3 days after the end of such treatments.

### Electro-acupuncture procedure

Patients in the acupuncture group will be treated at acupoints PC6 (Neiguan) and GV26 (Shuigou), as described in Table 2. The patient will be placed in a supine position. After disinfection of skin with 75% alcohol, the acupuncture needles (stainless steel, sterile and disposable, 30-guage in thickness, and 45 mm in length, Jiajian Medical Instrument Co. Std, Jiangsu, China) will be inserted into the respective acupoints. The needle will be inserted perpendicularly for PC6 at a depth of 20 mm. For GV26, the needle will be inserted towards the nose at a depth of 5–10 mm. The needle will be manipulated using twirling, lifting, thrusting, and mild reinforcing-reducing methods to promote Qi. Eye moisture or the presence of tears will be used as an indicator of Qi arrival since patients are not able to express their feelings. The electro-acupuncture apparatus electrodes will then be linked onto the needle handles of both sides of PC6. The electro-acupuncture apparatus will be set for disperse-dense waves at a frequency of 10/50 Hz and current of 1 mA. The needles will be retained for 60 min after electro-acupuncture for 30 min duration. Due to the specific location of GV26, only hand manipulation will be applied for 30 min.

**Table 2:** Description of acupuncture points

Acupoints	Descriptions
PC6: Neiguan	Locations: 2 cun above the transverse crease of the wrist, between the tendons of m. Palmaris longus and m. flexor radialis Indications: cardiac pain, mental disorder, epilepsy, insomnia, vomiting, hiccup, febrile disease Insertion depth: 20 mm
GV26: Shuigou	Locations: at the junction of the upper and middle third of the philtrum Indications: mental disorders, epilepsy, hysteria, coma, aplexy-faint Insertion depth: 5–10 mm

### Electrophysiological methods

To assess the patients’ consciousness level, event-related potentials (ERP) will be recorded from scalp electrodes placed on sites CZ and FZ according to the international 10–20 system, using the nose as reference. Mismatch negativity (MMN) will be produced using a pitch change in a repetitive auditory sequence. ERP will be recorded before electro-acupuncture starts, after the 10-day acupuncture is completed, on the day when the patient is discharged from ICU, and on the day of hospital discharge. MMN will be recorded at the same time of day for each measurement.

### Outcome measures

**Fig. 2:**
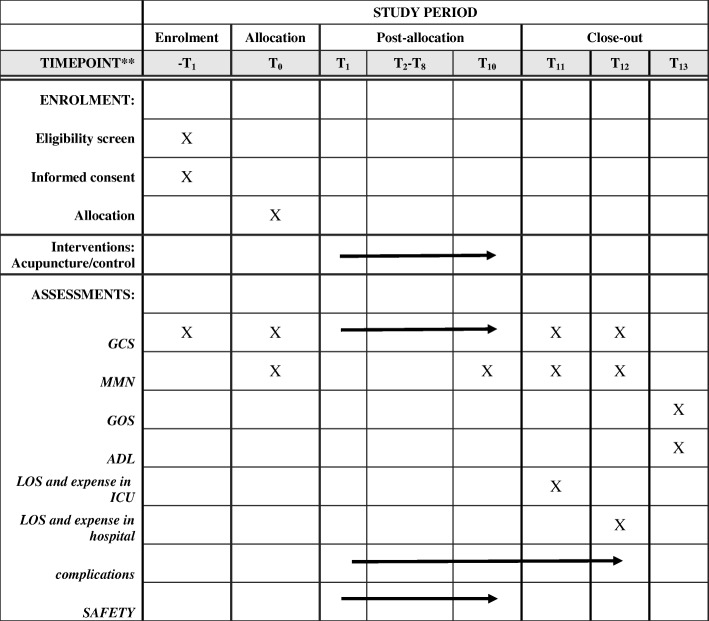
Schedule of enrollment, interventions, and assessments

The primary measurements for efficacy assessment will be changes in Glasgow Coma Scale (GCS) and MMN. Additionally, Glasgow Outcome Scale and Barthel Index of Activities of Daily Living at 3 months after the trauma will be compared between the two groups. Secondary measurements will be the length of stay in ICU and hospital, expenses in ICU and hospital, and the incidence of coma-related complications. Safety will be assessed by monitoring adverse events as well as changes in vital signs during the study (see Fig. 2 and Additional file 1: Standard Protocol Items: Recommendations for Interventional Trials (SPIRIT) Checklist).

### Safety monitoring

Patients’ legal representatives will be informed of potential adverse events from acupuncture prior to signing the consent form. Any adverse events, including acupoint hematoma, infection, and apostasis, will be recorded by the researcher. In case of severe adverse events occurring, acupuncture intervention will be ceased immediately and proper treatment will be provided. All severe adverse events will be reported to the principle investigator and the ethics committee within 48 h. Patients will be followed up for 1 month after the trial.

### Sample size calculation

The purpose of this study is to clarify the role of acupuncture in the consciousness recovery of patients with TBI. Therefore, GCS change will be used as an evaluation index. Changes in GCS before and after treatment in our pilot study were shown to be 2.27 ± 3.52 (*n* = 11) in the control group and 3.70 ± 2.16 (*n* = 12) in the acupuncture group. Sample size was calculated using the following formula:

$$ n={\left[\frac{\left({Z}_{\alpha /2}+{Z}_{\beta}\right)\sigma }{\delta}\right]}^2\left(\frac{1}{Q_1}+\frac{1}{Q_2}\right) $$

where *n* represents the number of samples required, *n* = *n*_1_ + *n*_2_, Q_1_ = *n*_1_/*n*, Q_2=_ *n*_2_/*n* with 80% power and significance level at 5% for a two-sided test. Thus, allowing for 10% of attrition, we will recruit 150 participants, with 75 in each group.

### Statistical analysis

SPSS 17.0 (IBM SPSS, Chicago, IL, USA) will be used to analyze the data. Quantitative data will be presented as mean ± SD. Single factor variance analysis will be used to compare the difference in heart rate, blood pressure, mean arterial pressure, respiratory rate, and saturation of pulse oxygen between the two groups. The χ^2^ test will be used to compare the incidence of complications and adverse events between groups. Measurement of neural function recovery, including GCS, Glasgow Outcome Scale, and activities of daily living, will be analyzed using rank sum-test. Longitudinal changes in MMN amplitude will be analyzed using a mixed model procedure. *P* values of less than 0.05 will be considered statistically significant.

### Quality control

In order to avoid possible heterogeneity in the measurement data and to ensure high-quality data results, the study data will be manually recorded on the case report form and uploaded to the database. The trial management team will control data quality through regular meetings and strict trainings to ensure that the acupuncturists, intensivists, neurosurgeons, and nurses are fully aware of the complete procedure. When the clinical trial begins, the principle investigator will supervise the trial to ensure that (1) the recruited patients are within the scope of the planned number; (2) all participants meet the inclusion criteria; and (3) all participants follow the clinical trial process. Auditing trial conduct will be performed every 3 months by investigators, intensivists, and surgeons.

## Discussion

TBI has become a severe socioeconomic issue due to its high mortality and morbidity. Patients surviving the original insult may remain in a comatose state for a considerable time. Effective and safe treatments to improve the outcome of patients with TBI are necessary. Despite acupuncture having long been used in the treatment of neurological diseases in China, its efficacy has attracted the interests of western medicine only in recent years [17] and several randomized controlled trials have been conducted to evaluate its potentials in clinical practice. However, the results are controversial due to the lack of high-quality studies, limited sample numbers, variation in acupuncture administration, and the possible placebo effects of acupuncture.

This study is designed to evaluate the efficacy and safety of electro- acupuncture in the consciousness recovery of patients with TBI. To reduce the possible bias, we have introduced strict quality control procedures, including randomization, blinding, adequate sample size, and proper control. Allocation concealment will be used to randomize the enrolled patients into acupuncture or control groups, and only the acupuncture doctors will know the patient’s group allocation. All data will be collected and analyzed by researchers aware of group allocation only when the trial has been completed. The initial GCS of patients will be set at 8 so as to largely reduce the placebo effects of acupuncture.

In this study, the acupuncture points of PC6 and GV26 have been selected based on Xingnaokaiqiao theory [18]. PC6 is an acupoint mainly used to improve cardiac function and increase cerebral perfusion. It has been proved, in an animal model, that acupuncture at PC6 can increase cardiac output as well as blood and oxygen supply to the brain, meliorating brain edema [19]. In addition, PC6 acupuncture may induce connections between cerebral cortex regions [20]. In China, GV26 is widely used in patients with stroke for prompt restoration of consciousness.

Mechanism studies using animal models have shown that electro-acupuncture may alleviate cerebral injuries via upregulation of transforming growth factor beta 1 [21]. Activation of large-conductance Ca^+^-activated K^+^ channels is also likely involved in the protective effects of GV26 [22]. Herein, in order to avoid variations in the acupuncture procedure, electro-acupuncture will be used to achieve good control of the depth, frequency, and duration of treatment.

ERP can provide important information on human brain functions. Among all the auditory ERP components, MMN is an automatic event related to brain response. MMN can be recorded in comatose patient and is considered a reliable predictor of awakening from coma, with a specificity of 91% [23]. When patients start to regain consciousness, there might be an enhancement of MMN amplitude prior to communicating with the environment. Herein, longitudinal changes in MMN responses will be investigated to identify early signs of recovery.

In conclusion, this study will provide solid evidence of the role of electro-acupuncture in the recovery of consciousness of patients with TBI.

### Trial status

Patient recruitment is ongoing. This is protocol version 20160701, version date is 2016.7.23. Recruitment was started in June 2017 and will be completed in June 2018.

## Additional file

Additional file 1: SPIRIT 2013 checklist for the study protocol. (DOC 127 kb)

## Abbreviations

DOC: Disorder of consciousness
ERP: Event-related potentials
GCS: Glasgow Coma Scale
MMN: Mismatch negativity
TBI: Traumatic brain injury
